# When user-centered design meets implementation science: integrating provider perspectives in the development of an intimate partner violence intervention for women treated in the United States’ largest integrated healthcare system

**DOI:** 10.1186/s12905-019-0837-8

**Published:** 2019-11-27

**Authors:** Sara B. Danitz, Shannon Wiltsey Stirman, Alessandra R. Grillo, Melissa E. Dichter, Mary Driscoll, Megan R. Gerber, Kristin Gregor, Alison B. Hamilton, Katherine M. Iverson

**Affiliations:** 1 Women’s Health Sciences Division of the National Center for PTSD (116B-3), VA Boston Healthcare System, 150 South Huntington Avenue, Boston, MA 02130 USA; 2 Dissemination and Training Division of the National Center for PTSD, VA Palo Alto Healthcare System, Menlo Park, CA USA; 3 Department of Psychiatry and Behavioral Sciences, Stanford University, Palo Alto, CA USA; 4 VA Center for Health Equity Research and Promotion (CHERP), Philadelphia, PA USA; 5 Temple University, School of Social Work, Philadelphia, PA USA; 6 Pain Research, Informatics, Multi-morbidities, and Education (PRIME), VA Connecticut Healthcare System, West Haven, CT USA; 7 Yale School of Medicine, New Haven, CT USA; 8 VA Boston Healthcare System, Boston, MA USA; 9 Boston University School of Medicine, Boston, MA USA; 10 VA Center for the Study of Healthcare Innovation, Implementation and Policy, Los Angeles, CA USA; 11 UCLA Department of Psychiatry and Biobehavioral Sciences, Los Angeles, CA USA; 12 Department of Psychiatry, Boston University School of Medicine, Boston, MA USA

**Keywords:** Intervention development, Provider perspectives, Treatment, Women veterans, Veterans Health Administration

## Abstract

**Background:**

Intimate partner violence (IPV) against women is a global health problem that is a substantial source of human suffering. Within the United States (US), women veterans are at high risk for experiencing IPV. There is an urgent need for feasible, acceptable, and patient-centered IPV counseling interventions for the growing number of women treated in the US’s largest integrated healthcare system, the Veterans Health Administration (VHA). Implementation science and user-centered-design (UCD) can play an important role in accelerating the research-to-practice pipeline. Recovering from IPV through Strengths and Empowerment (RISE) is a flexible, patient-centered, modular-based program that holds promise as a brief counseling intervention for women veterans treated in VHA. We utilized a UCD approach to develop and refine RISE (prior to formal effectiveness evaluations) by soliciting early feedback from the providers where the intervention will ultimately be implemented. The current study reports on the feedback from VHA providers that was used to tailor and refine RISE.

**Method:**

We conducted and analyzed semi-structured, key-informant interviews with VHA providers working in clinics relevant to the delivery of IPV interventions (*n* = 23) at two large medical centers in the US. Participants’ mean age was 42.6 years (SD = 11.6), they were predominately female (91.3%) and from a variety of relevant disciplines (39.1% psychologists, 21.7% social workers, 17.4% physicians, 8.7% registered nurses, 4.3% psychiatrists, 4.3% licensed marriage and family therapists, 4.3% peer specialists). We conducted rapid content analysis using a hybrid inductive-deductive approach.

**Results:**

Providers perceived RISE as highly acceptable and feasible, noting strengths including RISE’s structure, patient-centered agenda, and facilitation of provider comfort in addressing IPV. Researchers identified themes related to content and context modifications, including requests for additional safety check-ins, structure for goal-setting, and suggestions for how to develop and implement RISE-specific trainings.

**Conclusions:**

These findings have guided refinements to RISE prior to formal effectiveness testing in VHA. We discuss implications for the use of UCD in intervention development and refinement for interventions addressing IPV and other trauma in health care settings globally.

**Trial registration:**

ClinicalTrials.gov identifier: NCT03261700; Date of registration: 8/25/2017, date of enrollment of first participant in trial: 10/22/2018. Unique Protocol ID: IIR 16–062.

## Background

Intimate partner violence (IPV) against women is a global health problem because of its high prevalence, robust associations with poor health, and risk for premature death from homicide and suicide [1–3]. Within the United States (US), IPV is a particularly relevant issue for women who have served in the military [4, 5], with 19% of women veteran’s experiencing IPV in the past-year alone [6]. Women who experience IPV use more healthcare services [1, 7–9], a finding that is true across a number of service types within the Veterans Health Administration (VHA), the US’s largest integrated healthcare system [6, 10]. Consistent with some US-based recommendations for addressing IPV in health care settings [11, 12], VHA is in the process of implementing routing screening programs to identify women who experience IPV so that they can receive brief counseling and/or referrals to appropriate health and social services to address IPV.

Although differences in opinion exist about the impact of screening for IPV [13–19], there is a global concensus that healthcare providers should provide a first-line response and intervention [20]. However, less is known about *how* to effectively intervene to improve health outcomes following IPV disclosure in healthcare settings. While several countries have begun to address violence that women experience (e.g., in Canada: the Intervention for Health Enhancement After Leaving [iHEAL], which is a primary health care intervention for women recently separated from violent partners [21]; in Australia: Women’s Evaluation and Violence care in general practice (WEAVE [22];); and in South Africa, Brazil, Spain, India, and Lebanon [20]) there have been varying success rates. By and large the majority of countries can improve on their healthcare responses and policy [20] and methods for psychological intervention [23], despite that many private and public service settings, including the VHA in the US, have implemented or are in the process of implementing routine screening protocols to identify women who experience IPV. The current sub-study is part of a larger multi-phase study that seeks to help address this issue by determining the preliminary effectiveness of an intervention for women veterans experiencing IPV [24], which – should it prove to be effective – could help to bridge the gap in healthcare response following IPV disclosure.

Even integrated healthcare settings, such as VHA where there is a focus on enhancing the uptake of IPV screening into routine care, are challenged to provide well-defined guidance on implementation of interventions for women who disclose experiences of IPV. More specifically, there is a need to develop feasible, acceptable, patient-centered and effective interventions for women who experience IPV, especially for the unique needs of women veterans, and to ensure that these interventions move swiftly along the research-to-practice pipeline via implementation science and user-centered design.

User-centered design (UCD) is an approach to developing and refining products or programs and is rooted in soliciting early and regular feedback from the individuals in settings where the product will ultimately be implemented [25]. The field of UCD has developed over the past few decades (e.g., in settings of industrial design, cognitive psychology) and has recently been theorized to have utility for the development and implementation of psychosocial interventions [25]. UCD serves to improve the usability of products; this is especially pertinent to the development and refinement of interventions, and for the implementation of evidence-based treatments (EBTs) as it may serve to lessen the gap between research and practice in which there is a lack of uptake in delivering EBTs [26]. As such, utilizing a UCD framework in which feedback from providers is integrated throughout the development and refinement of the intervention holds promise for enhancing the usability and effectiveness of the intervention. Optimizing of the role of implementation science early on to accelerate the research-to-practice pipeline is essential to filling critical gaps in care, such as those which currently exist for women who experience IPV.

Recovering from IPV through Strength and Empowerment (RISE) is a flexible, patient-centered, trauma-informed, variable-length, modular-based intervention developed by clinicians and researchers in VHA that is currently being evaluated as part of a larger multi-phase study to determine its preliminary effectiveness for women veterans [24]. This brief counseling intervention was developed using rigorous user-centered treatment-development methods, including individual and focus group interviews with women veteran VHA patients and providers, followed by surveys with women veterans to further clarify women’s IPV-related counseling preferences and priorities [27–30]. RISE is an intervention based on empowerment, which is highly relevant for women who experience IPV [31, 32]. Based on this foundational research, RISE was developed to be flexible, with women veterans determining the intervention duration (between one and up to six 30–45 minute sessions) and choosing the topic to cover during each session. At the beginning of each RISE session, women are given a menu of options and encouraged to identify the area that would be most helpful to focus on that session to address their unique needs. RISE includes six topics addressing: A) safety planning, B) education on health effects of IPV, C) improving coping and self-care, D) enhancing social support, E) making difficult decisions, and F) connecting with resources; which align with recommended components of IPV interventions [33]. At the conclusion of each RISE session, in consultation with the provider, the woman is asked to set a goal related to the topic, and she is asked whether she would like to schedule an additional RISE session. RISE incorporates principles of Motivational Interviewing (MI) [34], an evidence-based approach designed to facilitate behavior change that is widely used to address numerous complex health issues, with some promise in addressing IPV [35, 36]. RISE was developed for delivery in integrated healthcare systems including in mental health outpatient clinics and primary care behavioral health. The intervention is designed to be user-friendly and accessible such that a range of providers, including psychologists and social workers, can implement it. The RISE manual is roughly 80 pages long, and separated into sections by modules, such that providers and patients can choose the section that is most relevant to them, without needing to go through each section sequentially [24].

RISE aims to improve women’s psychosocial functioning in domains that can reasonably expect to be enhanced in the context of a brief intervention for women experiencing recent and ongoing IPV. The proximal treatment targets include self-efficacy, valued action, patient activation, empowerment, and general psychological distress (e.g., depressive symptoms). Additionally, as part of a sub-study of this larger multiphase project, women veteran’s preferences for patient-centered outcomes were elucidated [37] to further enhance the likelihood that the RISE intervention will address women’s needs. Results demonstrated that women desired the following outcomes from RISE (and IPV intervention more broadly): increased empowerment, self-esteem, social support, IPV-related knowledge, valued action and goal setting [37]. While women veterans represent an important group of end-users of the RISE intervention, it is also critical that those whom are likely to provide the intervention give feedback that can enhance the likelihood that the intervention will be scalable in VHA.

The current sub-study is a part of a larger multi-phase project in the US designed to refine and formally evaluate RISE in preparation for an effectiveness trial within VHA. This sub-study focused on feedback from a wide array of providers regarding the acceptability and feasibility of RISE, and associated recommendations for refinements of content and context [38] in order to increase the likelihood of the usefulness, acceptability, and feasibility of the RISE intervention to VHA providers, the end-users, should RISE prove to be effective.

## Method

### Design

In this qualitative study, we conducted and analyzed semi-structured telephone interviews with VHA key informants working within two large VHA medical centers located in the New England region of the US (VA Boston Healthcare System and the VA Connecticut Healthcare System) during September–December 2017. The Institutional Review Boards (IRB) at both the VA Boston Healthcare System and the VA Connecticut Healthcare System provided ethics approval for this study.

### Participants

Eligible participants were individuals who were at least part-time VHA employees who worked in a relevant clinical capacity with female VHA patients. The study team identified potential participants based on their job titles and role/discipline listed through the hospitals’ e-mail system, and then sent recruitment e-mails to specific types of providers. This included those involved in coordinating relevant care for women, namely primary care physicians, social workers, psychologists, licensed marriage and family therapists, psychiatrists, and staff, including IPV Assistance Program Coordinators. (IPV Assistance Program Coordinators are mandated within VHA to assist with policies and procedures for addressing IPV within the local medical center and outpatient clinics, including coordinating referrals for women who disclose IPV and are interested in receiving interventions). As such, the study team used purposive sampling techniques to identify key informants whom work in clinics and roles relevant to the delivery of IPV interventions [39]. Project investigators contacted potential participants through e-mail to inform them of the study opportunity and invite them to participate. Those who expressed interest were scheduled for a 60-minute interview at their convenience. Prior to the interview, participants received the draft RISE manual to review if time permitted.

All participants provided verbal consent for the interview and for the audio-recording of the interview. Participants answered a few brief questions about their demographics and their VHA role(s) prior to beginning the interview.

### Approach

Study investigators with expertise in IPV, treatment development, and implementation science developed the interview guide, which contained questions about general impressions of the RISE intervention, any modifications needed to enhance feasibility, fit, effectiveness; and facilitators to and barriers of using RISE in routine care. Interview questions were semi-structured in nature, with a focus on eliciting provider perspectives on RISE, including potential contextual barriers and facilitators to RISE implementation [40, 41]. All interviews were conducted by the study project manager (SD) or principal investigator (KI), both of whom are Ph.D. level female psychologists with training in qualitative interviewing. The interviewer explained the purpose of the interviews, the rationale for the development of RISE, its underlying focus on empowerment, and its overall structure. After asking any initial questions, the participants had up to 20 minutes to review the RISE manual in order to get a better feel for the philosophy, structure, and content of the intervention. Interview questions assessed an array of factors relevant to the implementation of RISE within VHA. The semi-structured interviews were of varying lengths, with an average of approximately 43 minutes. Interviewers completed brief memos immediately after each interview. The team conducted interviews until variations in perspectives lessened and data became duplicative (i.e., saturation) [42]. All interviews were transcribed.

### Data analysis

The current study uses an established rapid content analysis approach to efficiently derive key findings from transcripts to tailor and refine the RISE manual [43]. As transcripts became available, they were reviewed and coded by members of the research team (one PhD-level researcher and two bachelor’s level research assistants, with supervision from the study PI) using top-level, deductive coding to capture the content of key topics from the interview guide. Using a hybrid deductive-inductive approach, the team added inductive codes as they identified emergent findings. We transferred summaries into matrices and used matrix analysis methods to identify key themes related to the RISE intervention and its implementation characteristics [44]. The matrices facilitated the discovery of relationships and patterns across participants, expediting synthesis and summary [44].

Initial implementation characteristics coding categories were derived from Proctor and colleagues’ taxonomy of implementation outcomes [45]. Further, researchers used Wiltsey Stirman and colleagues’ framework for modifications and adaptations of interventions to categorize end-user feedback into “content” versus “context” modifications [38]. According to this framework, content modifications include changes made to intervention materials or delivery, whereas context modifications include changes to the personnel who deliver the intervention or the format or setting of the intervention [38]. All members of the coding team coded the first 5 transcripts independently and then met for consensus to discuss any coding discrepancies and ensure coding agreement. Once agreement was reached, the next 18 transcripts were divided among the 3 coders, and 33% of the data (*n* = 6 transcripts) was double-coded and percent agreement was calculated in NVivo [46]. Percent agreement was 94.7% (ranging from 93.9–96.1%). Following the completion of coding, two members of the research team reviewed coding categories to independently identify sub-groups of key points from each of the coding categories using matrices that included key topics and exemplary quotes and interviewer notes from memos in order to capture all sources of data from the interviews.

## Results

A total of 23 key informants participated in this study (62% participation rate, with nearly equivalent representation across the two study sites). The mean age of participants was 42.6 years (SD = 11.6; range: 25–63) and the sample predominately identified as female (91.3%; *n* = 21). Participants had worked in VHA for a range of 2–25 years (mean = 8.6, SD = 6.9). Table 1 provides a breakdown of the sample by provider profession.

**Table 1:** Breakdown of Providers by Profession (*N* = 23)

Profession	Frequency	Percent %
Psychologist	9	39.1
Social worker	5	21.7
Physician	4	17.4
Registered Nurse	2	8.7
Licensed Marriage and Family Therapist	1	4.3
Peer Specialist	1	4.3
Psychiatrist	1	4.3

### Acceptability and appropriateness

Overall, findings indicated that RISE was highly acceptable to providers. They endorsed RISE as well-organized and user friendly, noting that, “*It’s very usable, I can really picture doing each of these activities with a veteran sitting in front of me. There’s something tangible for the veterans to take, to read, to visualize … [RISE] brings it to the veteran’s level where they are and normalizes it. It’s non- stigmatizing … it empowers the patient to be active.”* In particular, a number of providers endorsed appreciation for the modular style of the intervention, noting that the content feels manageable, and the format provides welcomed flexibility. Participants also indicated that they found the components of RISE, including its content and non-judgmental, empowering stance, to be highly relevant and appropriate to the unique needs of women dealing with IPV. One provider noted, “*I like that the script and the language of MI and the empowerment language mirrors that goal of helping a woman regain control over her own choices. I’ve found that it’s [autonomy/choice] taken away in the context of IPV and so I think that’s a critical element in addressing it.”*

### Relative advantage and facilitating factors

Providers noted the relative advantages of RISE compared to other related interventions (or lack thereof) or how they usually address IPV. A consistent finding was that providers perceived that the RISE intervention manual would help to facilitate provider comfort and confidence in addressing IPV, especially for those who have less familiarity with addressing IPV. Providers noted that the comprehensive nature of RISE is reassuring and its structured nature helps to ease provider anxiety, “*It’s helpful to have a structured approach for this. I know that many providers that I’ve worked with whether here or elsewhere feel a little bit more nervous about ongoing IPV because unlike many of the things that we deal with, the trauma isn’t in the past, it’s ongoing, so there’s an element of increased current risk that makes it a bit more stressful, and having clear tools for that I think can be very comforting for a provider.”* Another provider echoed that IPV, in particular, seems to be scary for providers, noting that even for those who are comfortable doing suicide assessments, they are reluctant to inquire about IPV for fear that they will hear something and *“not know what to do with it.”* As such, providers noted that RISE offers a structured approach that “*would make me feel much more confident to address [IPV] with a client.”*

When asked about how RISE compares with the way providers currently address IPV, providers noted that RISE is much more detailed and comprehensive, stating that *“it’s massively leaps and bounds ahead. In other settings I’ve had no guidance, so I was winging it.”* Overall these findings underscore the advantage of a structured yet flexible intervention like RISE in potentially facilitating provider self-efficacy and comfort in addressing IPV.

Providers also noted that the example scripts lend themselves to a conversational style with patients, which can help to make both the provider and patient alike feel at ease. This is particularly true for providers who may not address IPV as often; one provide expressed that *“If you’re someone who doesn’t work with [IPV] on a regular basis, but certainly might come across it, if I knew that I was going to have a meeting with someone for something unrelated to IPV, but I looked in their records and saw there was a history of it, I would pull out this manual and look at it and say ‘okay, here’s a way that I can approach this woman about this topic’ that will be constructive and helpful as opposed to who knows if I’m being helpful or not … .it’s reassuring, like here’s some steps, here’s some guides, there’s even an example of what you can say. I like that a lot, especially if you’re in uncharted territory.”* Another provider echoed that the manual is user friendly and accessible, *“I definitely think people would be open to using it. Among the good things about it is that it’s very brief … and the actual language within the manual and the sheets and everything are conversational and easy to read.”* Several additional advantages of RISE were noted, including that it is patient-centered (as opposed to a provider-driven protocol), transdiagnostic, proactive rather than reactive, feasible, and its flexibility accommodates women’s variable preferences and needs.

Participants also gave feedback regarding what types of providers and clinical contexts would be best suited to facilitate the delivery of RISE. Providers felt that RISE is a particularly good match for clinicians with training in mental health and psychosocial health issues. Such providers include psychologists (e.g., clinical, health psychologists), social workers, marriage and family therapists, as well as case managers and peer specialists. In addition, participants described IPV Assistance Program Coordinators as key referral sources for implementing RISE in VHA. In general, while MD practitioners (i.e., PCPs, psychiatrists) in the study endorsed that they were well suited to detect IPV or make referrals to RISE, they felt that mental health providers were better suited to deliver an intervention such as RISE, noting, “*it seems like there’s other people that are better suited to do it … it’s a better fit for somebody like a psychologist, peer specialist who does a lot of these more practical things.”* Providers recommended settings for RISE implementation including integrated women’s health primary care clinics, where social workers and mental health professionals are typically embedded within the clinic to address psychosocial health issues. Primary care mental health integration was noted as a particularly promising context. In addition, outpatient mental health (e.g., general mental health clinics, post traumatic stress disorder clinics), and case management were also mentioned.

### Barriers

Participants identified several potential barriers to implementing RISE. One participant noted that it may be difficult for providers to take a nonjudgmental, MI-informed stance with their patients who are experiencing IPV, noting, *“I think it’s really, really hard – providers really want to help, and they want to keep their patients safe, and as a provider, I’ve seen other providers have a really hard time with letting clients who are in abusive relationships stay in those relationships. So I think taking this really nonjudgmental frame and letting it be very patient-focused, patient-centered, and patient-directed, if it doesn’t go in the same direction that the provider thinks it should go in, I think that can create a really tough dynamic.”* Relatedly, providers noted the difficulties holding emotionally laden experiences. One provider expressed*, “I think there are people who are very uncomfortable sitting with trauma and violence and while I don’t think that’s specific to this protocol, there’s some folks who have very intense reactions to hearing about others experiencing trauma and abuse, so I could see them having difficulty from that standpoint.”*

Providers also identified barriers such as the limited time, resources, and space available in primary care and other busy clinical settings. In particular, the burden of other clinical responsibilities within primary care, coupled with large patient panels, make it difficult for some types of providers (e.g., primary care physicians, nurses) to have the time or wherewithal to provide components of an intervention such as RISE, “*I think people are really open to interventions, but honestly it just really comes down to time. If that wasn’t a factor and if people had more freedom to be able to provide interventions and try to help without being held hostage to a clinical reminder or to a performance indicator, I think people would be very open to doing something like this.”* Relatedly, the length of the manual and the time required to deliver it to patients were considered additional barriers of addressing IPV. Some participants expressed that an IPV intervention was not perceived as a priority by the clinic, the healthcare system, and/or the leadership. However, participants also perceived that the availability of a clearly defined and feasible intervention could help break down this barrier.

### Content modifications

Key informants suggested several content modifications. The most common request of providers emphasized the importance of providing a clearer protocol for assessing physical safety throughout the RISE intervention, even if the patient does not select the safety module to focus on. One provider suggested taking an approach similar to that in Dialectical Behavior Therapy (DBT) [47, 48] where safety is prioritized, “*The DBT approach of really being transparent up front in the initial introduction that I as a provider feel compelled to highlight if there’s something that I’m really worried about imminent safety, I’m going to really ask if we can discuss that piece.”* Several other informants also suggested a brief safety check-in during the beginning of each RISE session so that any concerns are prioritized in the session, whether it be through the Safety Planning module or weaved into other modules (e.g., Connecting with Resources). In addition, informants felt it was important that RISE providers understand that safety planning will look different depending on the woman’s unique situation, especially when the woman is considering leaving the relationship or is still in the relationship as opposed to having already left and feels safe. One provider suggested adding a provider tip to the manual to specify this, *“Add a tip before the safety planning saying that a safety plan likely looks different at these three sort of pivotal times in a relationship: if someone is deciding [whether or not to leave], if they’re planning to leave, and once they’ve left. Those are three very different phases, so a safety plan would look different if it’s tailored to one of those phases.”*

Other content modifications included suggestions for additional provider tips in the RISE manual about topics such as vicarious traumatization and the importance of RISE providers seeking consultation and self-care. Providers emphasized reasons this would be helpful, particularly for those with less experience with IPV and trauma, noting that *“It’s hard to hear this [IPV, trauma] sometimes … so I think it’s important to acknowledge – that hearing about trauma and relationship violence can be difficult, especially for folks who haven’t worked in trauma or in IPV before.”* Consultation offers a potential vehicle for seeking support; *“to be able to consult with others when you feel like there’s tension with the client or other difficulties arising, could be really normalizing and validating for providers as well.”* Informants suggested varying formats for consultation, including having a consultation phone line available to call into as needed, or having a structured consultation time set up following the initial dose of training. Additionally, providers requested a ‘cheat sheet’ or overview/summary page for each module for ease of provider use, as well as a patient manual that includes all of the handouts for the intervention. Informants also requested that SMART (i.e., Specific, Measurable, Attainable, Realistic, Time-based) goal terminology be embedded throughout the intervention, and that local resources be included in the manual in addition to the national resources.

### Context modifications

Researchers identified several context modifications. Informants requested that the RISE intervention be offered in different contexts in addition to individual counseling. A group setting format was frequently suggested, noting that “*being in a group with other women who have gone through or are going through what you’re going through can be invaluable, having that safe space makes it feel very normalizing for women.”* Others suggested settings for RISE include residential treatment programs as women may be able to engage fully in these safe and supportive contexts in which they are away from their abuser. In addition, a few providers recommended the potential for RISE to be delivered by telemedicine, which may break down some barriers to receiving care for some women, especially those who live far from VA.

Further, a modification that was frequently endorsed across providers related to training. Providers discussed the importance of a RISE-specific training, which could include role-plays to help providers acclimate to RISE’s structure and to observe and practice more difficult scenarios. Providers endorsed that an in-person workshop or training would be helpful, and could potentially be followed by ongoing consultation. Other informants suggested that brief trainings during clinical team meetings or via an online webinar training would be helpful for providers who are interested in learning the RISE intervention.

## Discussion

Like other health care systems in the US and across the globe, VHA is integrating mechanisms for identifying women who experience IPV and is in need of evidence-informed, acceptable, and feasible interventions to offer women veterans who desire IPV-related treatment. The current study solicited provider feedback to refine the RISE intervention and plan for implementation in VHA. Results revealed that providers perceived the intervention as highly acceptable, noting strengths and relative advantages including RISE’s nonjudgmental and empowering stance, patient-centered agenda, user-friendly structure, and its facilitation of both provider and patient comfort. Barriers to implementation were also identified, including provider discomfort with IPV and trauma, as well as limited time and resources to provide this intervention in a busy clinical setting, especially when IPV is not viewed as a priority health issue by a healthcare system. These findings have guided refinements to the RISE intervention both in terms of content and implementation characteristics. Study findings have implications for the utilization of UCD for other emerging interventions for IPV and for adapting or modifying existing IPV interventions.

A robust theme that researchers identified was the discomfort providers experience in inquiring about IPV. While inquiry about suicide and homicide risk is routinely done in both primary care and mental health settings, informants described reluctance to ask about IPV, in part due to the paucity of interventions for addressing IPV. This finding is supported by the literature that points to many barriers to routine IPV screening in primary care and mental health settings [49, 50]. As such, RISE has the potential to fill a critical gap in the field and to provide a framework for responding to IPV disclosure that goes beyond routine IPV screening. According to our findings, RISE may help to facilitate provider comfort with not only asking about, but also addressing IPV in a more comprehensive manner through delivering RISE or referring a female patient to RISE. This is important because provider self-efficacy is an important provider-level facilitator in addressing complex health issues, including IPV [30, 51]. Furthermore, key informants provided insights into the type of training that would be needed to help them develop a sense of self-efficacy in using RISE to address IPV.

In addition, as knowledge regarding IPV becomes more sophisticated and there continues to be calls to address IPV within healthcare systems [1], there is a need for a nuanced provider response to the disclosure of IPV. While well-meaning providers have likely urged those in unhealthy relationships to ‘just leave,’ such a simplified approach risks missing the important nuances of the complexities involved, including shared children, and the ambivalence often experienced by women who both care about a partner and are simultaneously being hurt by their partner. As such, interventions for women who have already left the violent relationship (e.g., the Intervention for Health Enhancement After Leaving [iHEAL; 21]), while highly important, are insufficient on their own. Interventions for IPV need to reflect these nuances, and provide a patient-centered framework that can meet women where they are – independent of whether or not women are ready, willing, or able to leave the unhealthy relationship at any given moment in time. As Hegarty and colleagues highlight, it is not sufficient to only assess for the safety of women and children when addressing IPV, but rather [interventions] “must also respect and promote the dignity of women, validate and understand the diversity of women’s experiences, withhold judgment about what a woman should do and when, and place ongoing support at the centre of the interaction between the woman practitioner,” [52]. Similar to the WEAVE intervention [36, 52], RISE provides a framework in order to do such, utilizing MI principles to work through ambivalence and empowering and encouraging women to make the decisions that are best for themselves and (when applicable) their families. Findings support that providers are appreciative of these qualities of the RISE intervention, providing language and a theoretical framework that meets these needs and thereby is perceived to build confidence and comfort in ways to holistically treat women dealing with IPV. In addition, findings of this study bolster support for utilizing MI principles to address IPV by suggesting their acceptability and appropriateness from the provider perspective [35, 36].

In line with a UCD framework, end-users and stakeholders identified themes of content and context modifications that serve to inform refinement to the RISE intervention. Themes that researchers identified (e.g., about safety, trainings, goal setting, patient materials) align well with other evidence-based psychotherapies, both with the VHA setting and beyond [e.g., 47, 48, 53, 54]. For example, the request for a handout only manual for patients in addition to the provider manual, as well as ongoing consultation following an in-person workshop or training, is similar to formats used to increase the feasibility of delivering Cognitive Processing Therapy (CPT) [53, 54] a widely used intervention for posttraumatic stress disorder that has been rolled out within VHA. Further, the request by participants for safety check-ins during each RISE session, independent of whether the patient identifies safety as the topic that she wants to focus on during the session, aligns with the DBT approach [47, 48] in which safety is inquired about and prioritized. Additionally, the suggestion to utilize SMART goal terminology in the manual will likely help to set women up for success in setting goals each session, such that women are identifying specific, attainable, and realistic goals that she can plan for and address between sessions, as opposed to lofty, unrealistic goals, which may lead to feelings of inadequacy. These modifications identified during provider interviews have informed refinements and improved the RISE intervention and its implementation characteristics.

While this study focused on providers working with veterans in the US’s largest integrated healthcare system, findings may be broadly applicable to other countries that provide healthcare to former service members and veterans, such as the Veterans Affairs Canada, the Department of Veterans Affairs in Australia, and the Service Personnel and Veterans Agency in the United Kingdom [55]. For example, issues raised by providers in this study regarding vicarious traumatization, the importance of providers treating trauma and IPV seeking consultation and self-care, are not specific to providers in the US, and are broadly applicable to providers working with veterans and/or trauma globally. Consultation offers a possible venue for providers to seek support from peers that may help to offset some of the impact of vicarious traumatization.

Further, findings may be applicable for women’s health providers who are addressing IPV and trauma broadly. For example, the finding that having a structured approach to responding to IPV, which includes example scripts that could facilitate provider comfort in addressing IPV, may likely be transferrable for providers working with IPV across other settings and contexts. Additionally, the barrier identified that providers may have a difficult time utilizing a nonjudgmental stance and MI principles when responding to women who plan to remain in violent relationships is likely applicable broadly. Utilizing MI principles, and drawing from the Transtheoretical Model of Behavior Change [56] to understand the stages of change that a patient is currently in, as Hegarty and colleagues do in their WEAVE intervention in Australia [52], is a helpful tool in ensuring that therapy does not become a ‘tug of war’ between provider and patient, and that providers can meet patients where they are at in terms of readiness for change. This study supports utilization of Wiltsey Stirman and colleagues’ framework when conducting UCD research throughout the treatment development and evaluation for identifying content, context, and training modifications to interventions [38]. The framework was useful for organizing and characterizing the variety of suggestions for refinement, and may be useful in guiding or supporting intervention development or pre-implementation efforts to adapt other interventions to increase their relevance and feasibility in routine care delivery. Thus, this research may prove useful to other efforts that could benefit from established frameworks and methodologies for enhancing interventions and incorporating research on implementation characteristics that will likely increase the adoption, acceptability, and feasibility of the interventions by the intended end-users.

Several limitations exist in the present study. Providers interviewed in this study work at VA Healthcare Systems in the New England region of the US, and therefore findings may be specific to these regions and healthcare systems. As noted above, however, several findings are particularly applicable to other settings and to women’s health providers more generally. Nearly 40% of the sample were psychologists, and as such other professions may be under represented in the current study, possibly due to that physicians busy schedules may reduce engagement in voluntary research studies. Further, the current study did not formerly assess for provider’s level of training in or experience with IPV, which may have influenced results. Additionally, it is possible that other undue influences (e.g., social desirability) may have impacted provider response to study team members about the RISE intervention. Further, RISE was developed specifically to target the needs of women veterans who have experienced IPV, and while its principles likely could apply to some other settings in the US and across the globe, our findings may not be broadly applicable to implementing RISE in countries where healthcare resources are limited.

Future research should examine the RISE intervention in practice using hybrid study designs [57], in order to both examine the effectiveness of the intervention on psychosocial outcomes (i.e., clinical outcomes) as well as to provide evidence for the acceptability and feasibility (i.e., implementation outcomes) anticipated by providers in the current study. Trainings and workshops for the RISE intervention should be further developed and examined based on the feedback provided in this study. Calls for role-plays, web-based training, and ongoing consultation are aspects of implementation that may be particularly applicable to other IPV interventions and other health care systems across the globe.

An additional area for research involves determining the preferred settings for RISE implementation and dissemination. While providers in the current study expressed hesitancy about implementing RISE in primary care settings due to time and resource constraints, more research is needed to determine whether RISE can be effectively implemented in integrated primary care settings that include co-located mental, behavioral, or social health providers, such as social workers and psychologists, as part of the primary care medical home (referred to as Patient Aligned Care Teams in VHA) and primary care mental health integration. Primary care may be a setting that women are more willing to access given that it is not associated with the same stigma as mental health clinics, and many women veterans are already followed in primary care routinely. Further, RISE’s brief sessions (30–45 minutes) and short-term nature (one and up to six sessions) may make it an ideal intervention to implement in the fast-paced primary care setting. Further, providers noted IPV Coordinators as particularly well-suited for delivering RISE with women veterans, given that all VHA medical centers are required to have an identified IPV Assistance Program Coordinator to assist with policies and procedures for addressing IPV, as well as to accommodate ‘warm handoffs’ and referrals for women who disclose IPV and are interested in receiving interventions. In addition, if effective in an individual format, more research is needed to examine RISE in different formats, such as in a group setting, which would allow for social support and normalization among other women who have experienced IPV, but may not allow for as much individual autonomy in selecting the module most relevant to a patient’s specific needs.

## Conclusions

IPV against women is a critical population health issue. There remains a need to develop and evaluate interventions to address IPV, especially among vulnerable population such as women who have served in the military. This study described an example of applying UCD and implementation science principles to inform refinement to the first IPV intervention of its kind for women veterans in the US, for which there is a great need. We are hopeful that by eliciting provider feedback early on in intervention development and by incorporating current findings into intervention refinement prior to formal effectiveness evaluation, we will accelerate the timeline for implementing RISE into routine care, should it prove effective. We are hopeful that these methods and findings can be applied broadly to other settings globally in which providers are working with women to address IPV and trauma.

## Data Availability

The datasets generated during and/or analyzed during the current study are not publicly available due to Human Studies protections placed upon them by the Boston VA Healthcare System and VA Connecticut Healthcare System Institutional Review Boards. Data are available from the authors upon reasonable request, which would also involve obtaining permission from the VA Bostton Healthcare System Institutional Review Board and VA Connecticut Healthcare system Institutional Reivew Board.

## Abbreviations

CPT: Cognitive Processing Therapy
DBT: Dialetical Behavior Therapy
EBTs: Evidence-based treatments
iHEAL: Intervention for Health Enhancement After Leaving
IPV: Intimate partner violence
IRB: Institutional Review Board
MI: Motivational Interviewing
RISE: Recovering from IPV through Strengths and Empowerment
SMART: Specific, Measurable, Attainable, Realistic, Time-based
UCD: User-centered design
US: United States
VA: Veterans Affairs
VHA: Veterans Health Administration
WEAVE: Women’s Evaluation and Violence care in general practic
